# Renal function is associated with blood neurofilament light chain level in older adults

**DOI:** 10.1038/s41598-020-76990-7

**Published:** 2020-11-23

**Authors:** Shoshin Akamine, Noriko Marutani, Daisuke Kanayama, Shiho Gotoh, Riki Maruyama, Kanta Yanagida, Yukako Sakagami, Kohji Mori, Hiroyoshi Adachi, Junji Kozawa, Norikazu Maeda, Michio Otsuki, Takaaki Matsuoka, Hiromi Iwahashi, Iichiro Shimomura, Manabu Ikeda, Takashi Kudo

**Affiliations:** 1 Department of Mental Health Promotion, Osaka University Graduate School of Medicine, Suita, Osaka Japan; 2 Health and Counseling Center, Osaka University, Toyonaka, Osaka Japan; 3 Department of Psychological Health Promotion, Osaka University Graduate School of Medicine, Suita, Osaka Japan; 4 Department of Psychiatry, Osaka University Graduate School of Medicine, Suita, Osaka Japan; 5 Department of Metabolic Medicine, Osaka University Graduate School of Medicine, Suita, Osaka Japan; 6 Department of Diabetes Care Medicine, Osaka University Graduate School of Medicine, Suita, Osaka Japan; 7 Department of Metabolism and Atherosclerosis, Osaka University Graduate School of Medicine, Suita, Osaka Japan; 8 Department of Internal Medicine, Toyonaka Municipal Hospital, Osaka, Japan

**Keywords:** Neurodegenerative diseases, Kidney diseases, Type 2 diabetes, Biomarkers, Neurology

## Abstract

Neurofilament light chain (NfL) is a novel biomarker of neurodegenerative diseases. It is detectable in the peripheral blood, allowing low-invasive assessment of early signs of neurodegeneration. The level of NfL gradually increases with age; however, what other factors affect it remains unclear. The present study examined the association between blood NfL level and renal function among healthy participants undergoing a health check (n = 43, serum NfL) and patients with diabetes mellitus (n = 188, plasma NfL). All participants were 60 years of age or older; none were diagnosed with dementia. In each group, levels of blood NfL and serum creatinine significantly correlated (coefficient r = 0.50, 0.56). These associations remained statistically significant even after adjustment for age, sex, and body mass index. These findings indicate that blood NfL level might be partially affected by renal function. We recommend measuring renal function for a more precise evaluation of neuroaxonal damage, in particular, among older adults.

## Introduction

An ability to detect and track the course of a neurodegenerative disease is paramount to research and clinical practice, allowing evidence-based decisions regarding treatment initiation and outcome evaluation. Neurofilament light chain (NfL) is a neuronal cytoskeletal protein primarily expressed in large-caliber myelinated axons^1,2^. It has recently gained attention as a biomarker of neuroaxonal damage. Ultrasensitive immunodetection methods enabled reliable quantification of NfL in not only cerebrospinal fluid (CSF) but also serum and plasma^3^. Previous studies have reported an association between CSF and blood concentrations of NfL, suggesting both reflects shared physiological and pathological mechanisms within the central nervous system (CNS)^4–7^. Blood-based measurement of NfL is a promising low-invasive method of assessing various neurologic conditions, including multiple sclerosis, amyotrophic lateral sclerosis, frontotemporal dementia, Alzheimer’s disease, traumatic brain injury, and HIV-1 associated neurocognitive disorders^5,8–14^.

To effectively use NfL as a biomarker of neurodegeneration for research purposes, accurate identification of potentially confounding factors is required. A recent study has reported an association between plasma NfL level and body mass index (BMI) or blood volume^15^. However, the evidence is inconsistent on whether there is an association between blood NfL level and renal function^16,17^. Blood NfL and renal function both change with age, but to our knowledge, no study had investigated the association between plasma NfL level and renal function adjusted for age.

The present study investigated the relationship between blood NfL concentration and renal function in two groups separately and first assessed whether this relationship is independent of age, sex, and BMI.

## Methods

### Study participants

This study was approved by the research ethics committees of Osaka University Health and Counseling Center (HaCC), and Osaka University Hospital. All participants provided written informed consent, and all research was performed in accordance with relevant guidelines and regulations.

In the Health-Checkup (HC) group, 158 study participants were employees of Osaka University (aged > 60 years) who received an annual medical exam between April 2016 and March 2017 at the HaCC. Individuals with HbA1c (hemoglobin A1c) > 6.5% were excluded from the analysis. Serum samples were collected, processed, aliquoted, and frozen for later use. Samples underwent the freeze–thaw procedure only once. We randomly selected 44 out of 158 samples for NfL measurement and analysis, based on sample size calculation described below.

In addition, we recruited 198 outpatients with type 2 diabetes mellitus (DM) who visited the Department of Metabolic Medicine of Osaka University Hospital, Osaka, Japan, from July 2018 to June 2019. Clinical data or blood samples were not available for 12 individuals. Thus, a total of 188 participants were included in this study.

### Measurements of serum creatinine and estimation of glomerular filtration rate

We examined the relationship between blood NfL and serum creatinine levels in the HC group, since serum creatinine level reflects renal function.

In both groups, serum creatinine and urea nitrogen levels were acquired from the existing medical records. The estimated glomerular filtration rate (eGFR) was calculated using the following formula developed and validated in the Japanese population (1), which uses age and sex as surrogates for creatinine generation^18^:

1 $$ {\text{eGFR }}\left( {{\text{mL}}/{\text{min}}/1.{\text{73m}}^{2} } \right){\mkern 1mu}  = {\mkern 1mu} 194{\mkern 1mu}  \times {\mkern 1mu} {\text{serum creatinine}}_{{[{\text{mg}}/{\text{dL}}]}} ^{{ - 1.094}}  \times {\text{ age}}_{{[{\text{years}}]}} ^{{ - 0.287}} \left( { \times {\mkern 1mu} 0.{\text{739 for female}}} \right). $$

### Measurements of serum neurofilament light chain

Serum or plasma NfL concentration was measured in duplicate with ultrasensitive single-molecule array (Simoa) platform, using Simoa NF-Light Advantage kits on a Simoa HD-1 Analyzer instrument (Quanterix Corporation), according to the manufacturer’s instructions. The lower limit of quantification for the NfL concentration was 0.466 pg/mL. Samples with either fatal measurement errors or coefficients of variance (CV) > 20% were excluded from the analysis. In all batches, two types of quality-control samples provided in the kit were measured in duplicate, ensuring measurement validity.

In the HC group, one sample was excluded due to fatal measurement errors in both replicates. The remaining 43 samples were all included in the analysis. The mean CV of duplicates in this group was 5.3%. In the DM group, all 188 samples were included in the analysis. The mean CV of duplicates in this group was 3.6%.

### Statistical analysis

The sample size in the HC group was calculated by G*Power version 3.1.9.6^19^, using the following parameters: r(correlation coefficient) = 0.4, α = 0.05, Power(1 − β) = 0.8, which intended to detect correlation with medium-to-large effect size.

Due to the non-normal distribution of the data, blood NfL and creatinine values were log-transformed before statistical calculations were performed (Fig. S3). The Pearson correlation was used to calculate the strength of the association between variables of interest. A Student’s t-test was used to compare blood NfL levels between sexes. Using blood NfL as a dependent variable, age, sex, BMI and serum creatinine were included in a linear regression analysis. Because age, sex, and serum creatinine are used for the eGFR calculation, multivariate adjustment by these variables was not performed when analyzing the association between eGFR and blood NfL level to avoid collinearity problems.

All tests were two-sided. Statistical significance was set as *p* < 0.05. JMP Pro version 14.3.0 (SAS Institute Inc.) was used for statistical analysis.

## Results

Participants’ demographic characteristics are presented in Table 1. All participants were aged above 60 years.

**Table 1:** Demographic data of the two groups examined in this study.

	HC group (n = 43)	DM group (n = 188)
**Demographics**		
Age, y, mean (SD)	62.2 (2.26)	75.2 (6.06)
Sex, n, m/f	32/11	103/85
**Clinical characteristics**		
Height, cm, mean (SD)	165.4 (7.83)	160.0 (9.00)
Weight, kg, mean (SD)	65.4 (9.76)	61.3 (11.1)
BMI, kg/m^2^, mean (SD)	23.9 (2.68)	24.0 (3.61)
Serum creatinine, mg/dL, median (IQR)	0.80 (0.70–0.89)	0.83 (0.68–1.09)
UN, mg/dL, median (IQR)	13.5 (11.9–16.1)	18.0 (15.0–22.0)
eGFR, mL/min/1.73 m^2^, median (IQR)	69.9 (65.0–80.3)	59.5 (48.6–71.3)
HbA1c, %, median (IQR)	5.6 (4.2–5.8)	7.1 (6.6–7.7)
Serum NfL, pg/mL, median (IQR)	13.4 (11.7–16.5)	
Plasma NfL, pg/mL, median (IQR)		20.3 (14.6–30.6)

*SD* standard deviation; *IQR* interquartile range; * BMI* body mass index; * UN* urea nitrogen; * eGFR* estimated glomerular filtration rate; *NfL* neurofilament light chain; *HbA1c* hemoglobin A1c.

### Association between blood NfL and serum creatinine levels

The two groups were separately assessed for an association between blood NfL level and serum creatinine level.

**Figure 1:**
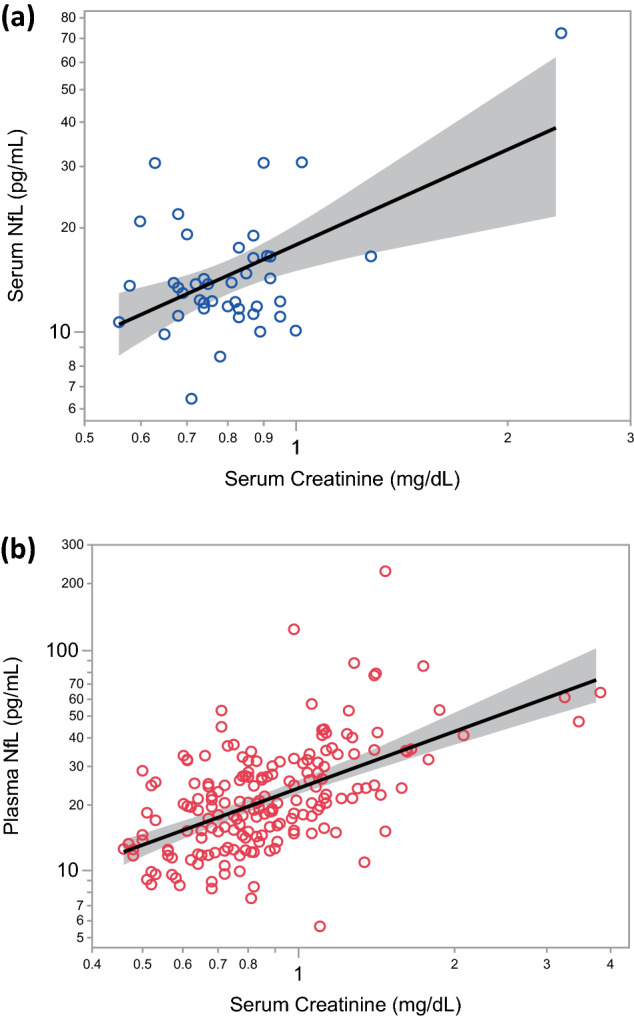
A positive correlation between blood NfL and serum creatinine levels in the HC group (**a**) and the DM group (**b**). NfL: neurofilament light chain.

Serum NfL was significantly correlated with serum creatinine levels in the HC group (r = 0.50, 95% confidence interval [CI]: 0.23, 0.69, *p* = 0.0007; Fig. 1a).

In the DM group, plasma NfL level significantly correlated with serum creatinine level (r = 0.56, 95% CI: 0.45, 0.65, *p* < 0.0001; Fig. 1b), consistent with the HC group findings.

### Association with age, sex, and BMI

As associations between blood NfL level, age, and BMI have been previously reported^5,10,15^, we adjusted our regression analysis of the association between blood NfL and serum creatinine levels for age, sex, and BMI (Table 2).

**Table 2:** Univariate and multivariate linear regression analyses of blood NfL level and age, sex, BMI and serum creatinine.

Variable	Univariate analysis		Multivariate analysis	
	Standardized β	*p*-value	Standardized β	*p*-value
**HC group (n = 43, serum NfL)**				
Age	0.110	0.48	0.134	0.33
Sex [M]	0.091	0.56	− 0.212	0.17
BMI	− 0.040	0.80	− 0.238	0.10
Serum creatinine	0.498	0.0007	0.660	0.0001
**DM group (n = 188, plasma NfL)**				
Age	0.400	< 0.0001	0.320	< 0.0001
Sex [M]	0.121	0.10	− 0.236	0.0001
BMI	− 0.231	0.0014	− 0.211	< 0.0001
Serum creatinine	0.560	< 0.0001	0.656	< 0.0001

See also supplementary Figs. 1 and 2.

*BMI* body mass index; *NfL* neurofilament light chain.

In the HC group, serum NfL level was not significantly associated with age (*p* = 0.48) or BMI (*p* = 0.80). No significant differences in mean serum NfL levels were observed between sexes (*p* = 0.56). The association between serum NfL and serum creatinine levels remained significant after adjusting for age, sex, and BMI (*p* = 0.0004).

In the DM group, there was a significant correlation between plasma NfL level and age (r = 0.40, 95% CI: 0.28, 0.51, *p* < 0.0001), but not between serum creatinine level and age (r = 0.07, 95% CI: − 0.07, 0.21, *p* = 0.30). BMI was negatively correlated with plasma NfL level (r =  − 0.23, 95% CI: − 0.36, − 0.09, *p* = 0.0014). No significant differences in mean plasma NfL levels were observed between sexes (*p* = 0.10). After adjusting for age, sex, and BMI, the association between plasma NfL and serum creatinine levels remained significant (*p* < 0.0001).

### Estimated renal function and blood NfL level

**Figure 2:**
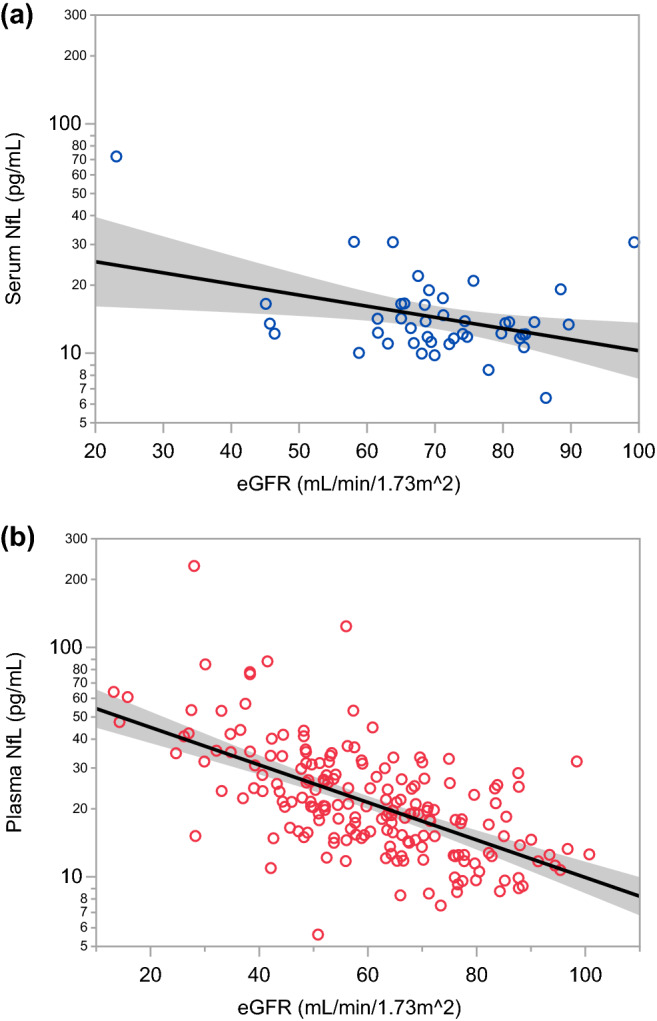
A positive correlation between blood NfL level and eGFR in the HC group (**a**) and the DM group (**b**). NfL: neurofilament light chain, eGFR: estimated glomerular filtration rate.

A moderate correlation between blood NfL level and eGFR was observed in the HC (r =  − 0.446, 95% CI: − 0.652, − 0.178, *p* = 0.0019) and DM group (r =  − 0.473, 95% CI: − 0.578, − 0.355, *p* < 0.0001), indicating that approximately 20% of the variability in blood NfL levels can be explained by renal clearance (Fig. 2).

## Discussion

This cross-sectional study of two different populations showed a positive correlation between blood NfL and serum creatinine levels in each group, indicating that blood NfL level may be partially influenced by renal function. Moreover, we have shown that blood NfL level in the DM group significantly correlated with age, but not with sex, a finding consistent with previous reports^5,10,15^, whereas the HC group analysis was underpowered to detect some of the association due to narrow age distribution and focused on detecting moderate-to-strong association. In the present study, the correlation between blood NfL and serum creatinine levels remained significant after adjustment for age, sex, and BMI. Estimated GFR calculated from serum creatinine, age, and sex was also significantly correlated with blood NfL in each group. These findings support the hypothesis about a negative correlation between blood NfL levels and renal function.

Two reports assessed relationships between blood NfL level and renal function. Consistent with the present data, Korley et al. have reported poor renal function as associated with a higher serum NfL level in a single cohort of patients with type 2 diabetes (mean age = 62.8 years)^16^. Concurrently, Hermansson et al. reported that plasma NfL level is not correlated with serum creatinine levels among patients with HIV infection (median age = 41 years)^17^. This inconsistency may be explained by between-study differences in age distribution, suggesting that the relationship between blood NfL and renal function becomes more evident among older adults. Not only in patients with type 2 diabetes, but the current study also showed that the association between blood NfL and renal function may be present in healthy individuals.

While the mechanism underlying how kidney function affects blood NfL dynamics are not known, there are some possibilities. One hypothesis is that blood NfL is cleared by the kidneys. This hypothesis suggests there is a risk of overestimating the extent of neuroaxonal damage among adults with low renal function.

Another possible mechanism incorporates the other functions of the kidneys. The decline of renal function leads to low levels of erythropoietin (EPO) and active vitamin D, both of which are primarily synthesized in the kidney^20^. EPO receptors are expressed in not only the bone marrow but also the brain^21–23^, and EPO is reported to have neuroprotective effects^24^. Active vitamin D is also reported to have neuroprotective effects^25^, and its deficiency is associated with various neuropsychological diseases such as depression, Alzheimer’s disease, and cerebrovascular dementia^26–29^. Blood levels of EPO and active vitamin D may link renal function and neuronal damage.

Although further investigations are required to determine the underlying mechanism, studies involving blood NfL level should assess renal function with serum creatinine or eGFR. When involving an older adult population, in particular, such an approach can help achieve precise assessment of neuroaxonal damage within the CNS.

This study has several limitations. Since we did not measure CSF NfL levels, it remains unknown whether NfL dynamics within the CNS are related to renal function. However, blood NfL level has been reported to strongly correlate with the CSF NfL level^4–7^. Because the study group comprised participants aged 60 years and older, these findings are not necessarily generalizable to all age groups. The sample size of the HC group was underpowered to detect a correlation coefficient smaller than 0.4 and the age distribution was narrow due to the Japanese system of mandatory retirement at the age of 65 while previous studies reported the correlation coefficient between age and blood NfL to be 0.32–0.59^30–32^. The association of blood NfL with renal function is confirmed in two distinct clinical groups, but the probability of confounding by comorbidities of diabetes such as peripheral neuropathy cannot be excluded, because we did not analyze the association with severities of comorbidities of diabetes in the DM group. Finally, because the present study used exclusively cross-sectional data, a causal relationship between blood NfL level and renal function was not assessed.

In summary, we observed a moderate correlation between blood NfL level and renal function among older adults who were healthy or had type 2 diabetes, which was independent of age, sex, and BMI. When analyzing blood NfL level as a marker of neurodegeneration, we recommend measuring renal function to assess the extent of neuroaxonal damage more precisely.

## Supplementary information

Supplementary Information.

## Data Availability

The data used in this study are available from the corresponding author on reasonable request.
