# Supplementary material for: Renal function is associated with blood neurofilament light chain level in older adults
Source: Sci Rep. 2020 Nov 23;10:20350. doi: 10.1038/s41598-020-76990-7 (PMC7683708; doi:10.1038/s41598-020-76990-7)
Supplement: Supplementary file 1 — Supplementary Information. [file 41598_2020_76990_MOESM1_ESM.pdf]

Renal Function is Associated with Blood Neurofilament Light Chain Level in Older Adults

Shoshin Akamine[1,2], Noriko Marutani[2,3], Daisuke Kanayama[1,2,4], Shiho Gotoh[4], Riki Maruyama[1,2], Kanta Yanagida[1,2], Yukako Sakagami[1,2], Kohji Mori[4], Hiroyoshi Adachi[1,2,4], Junji Kozawa[5,6], Norikazu Maeda[5,7], Michio Otsuki[5], Takaaki Matsuoka[5], Hiromi Iwahashi[5,6,8], Iichiro Shimomura[5], Manabu Ikeda[4], Takashi Kudo[1,2,4]

Department of Mental Health Promotion, Osaka University Graduate School of Medicine, Suita, Osaka, Japan.

Health and Counseling Center, Osaka University, Toyonaka, Osaka, Japan.

Department of Psychological Health Promotion, Osaka University Graduate School of Medicine, Suita, Osaka, Japan.

Department of Psychiatry, Osaka University Graduate School of Medicine, Suita, Osaka, Japan.

Department of Metabolic Medicine, Osaka University Graduate School of Medicine, Suita, Osaka, Japan.

Department of Diabetes Care Medicine, Osaka University Graduate School of Medicine, Suita, Osaka, Japan.

Department of Metabolism and Atherosclerosis, Osaka University Graduate School of Medicine, Suita, Osaka, Japan.

Department of Internal Medicine, Toyonaka Municipal Hospital, Osaka, Japan.

Supplementary Information

Supplementary Figure 1. Scatterplot between blood NfL level and age (a), sex (b) in the HC group. NfL: neurofilament light chain.

(a)

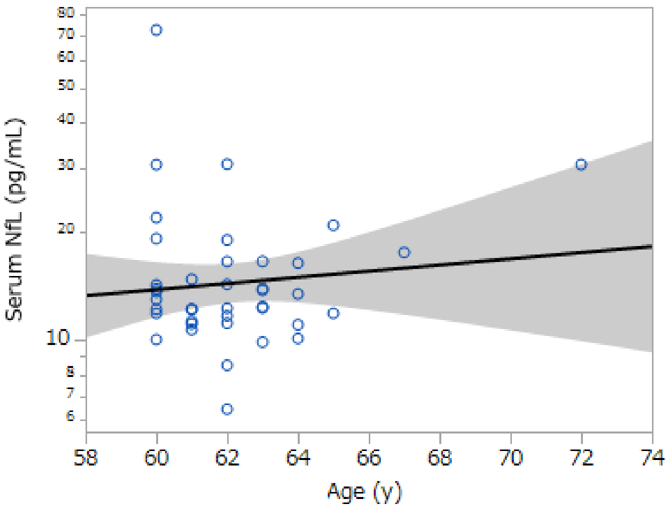

(b)

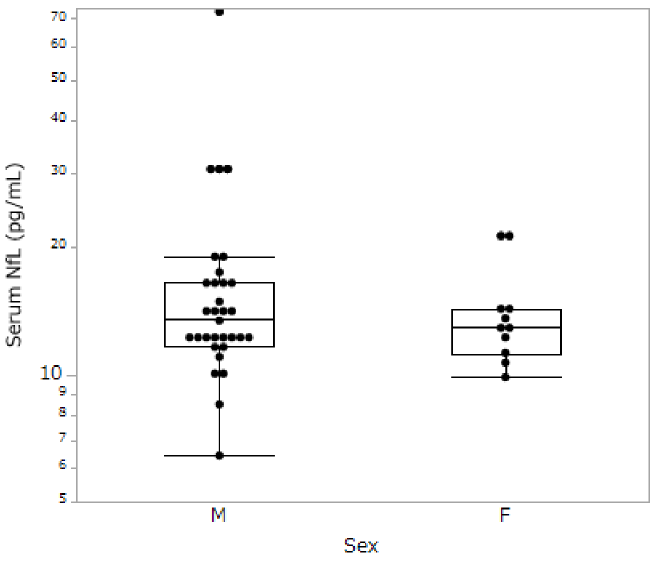

Supplementary Figure 2. Scatterplot between blood NfL level and age (a), sex (b) in the DM group. NfL: neurofilament light chain.

(a)

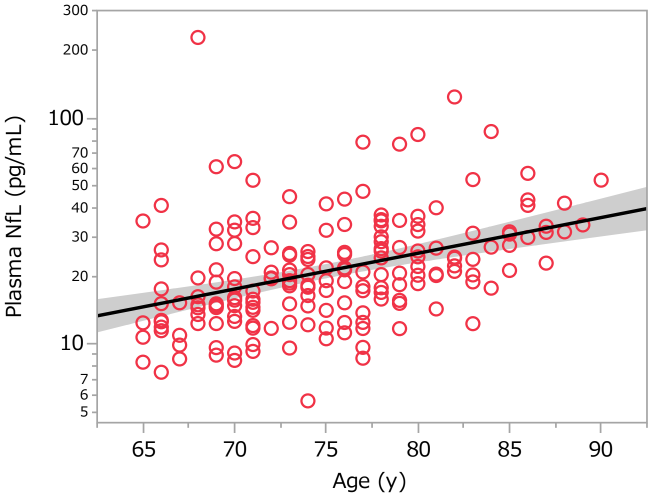

(b)

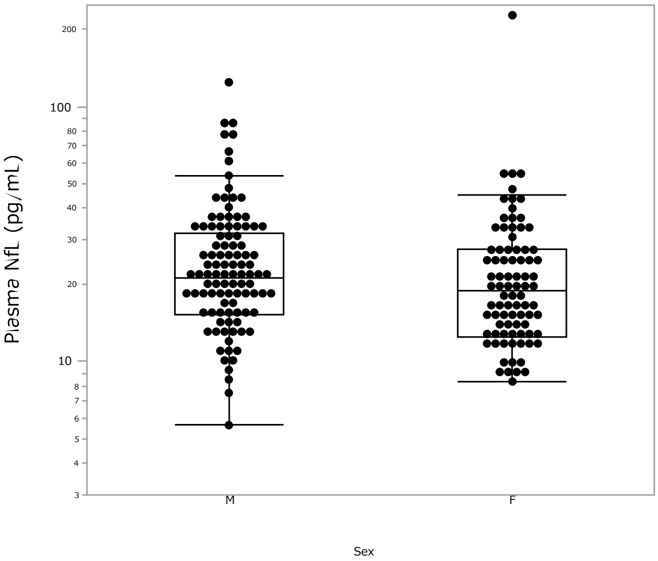

Supplementary Figure 3. Q-Q plot before and log-transformation of serum creatinine level and blood neurofilament level in the (a) HC group and (b) DM group.

(a)

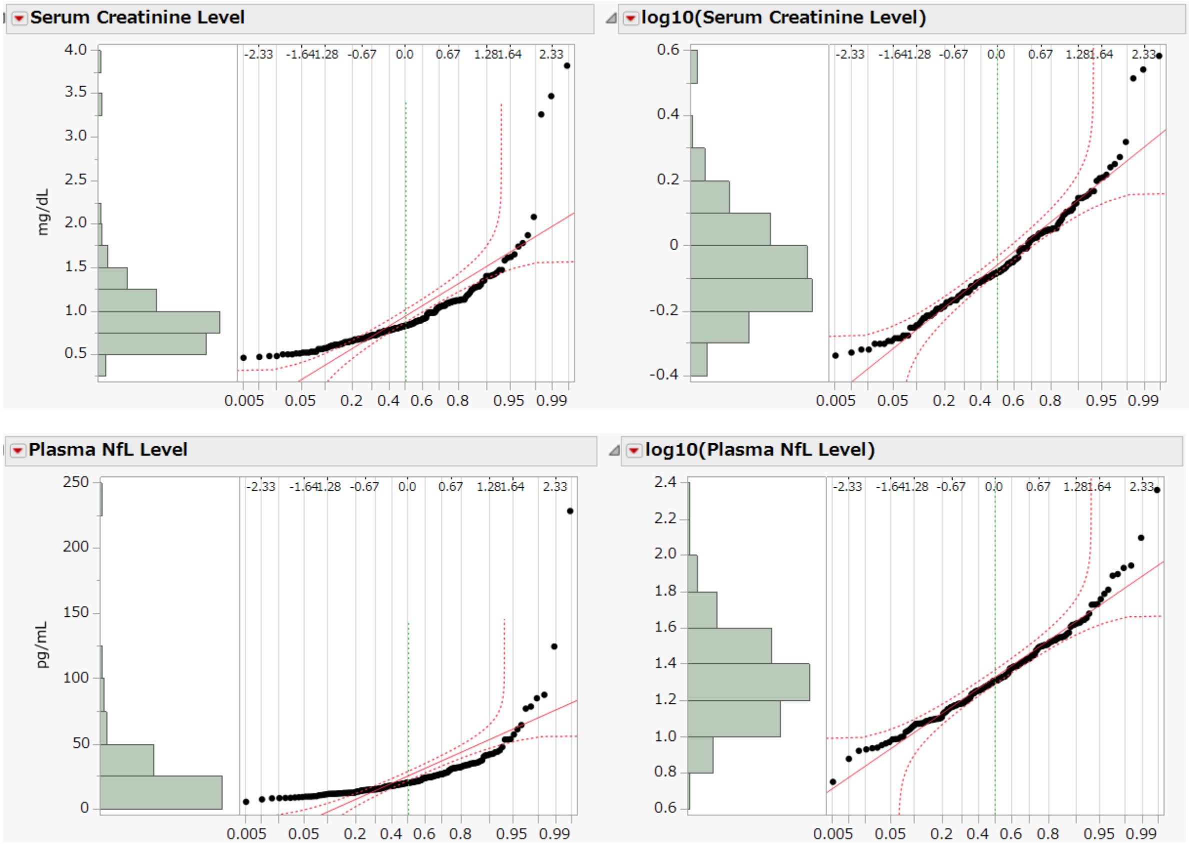

(b)

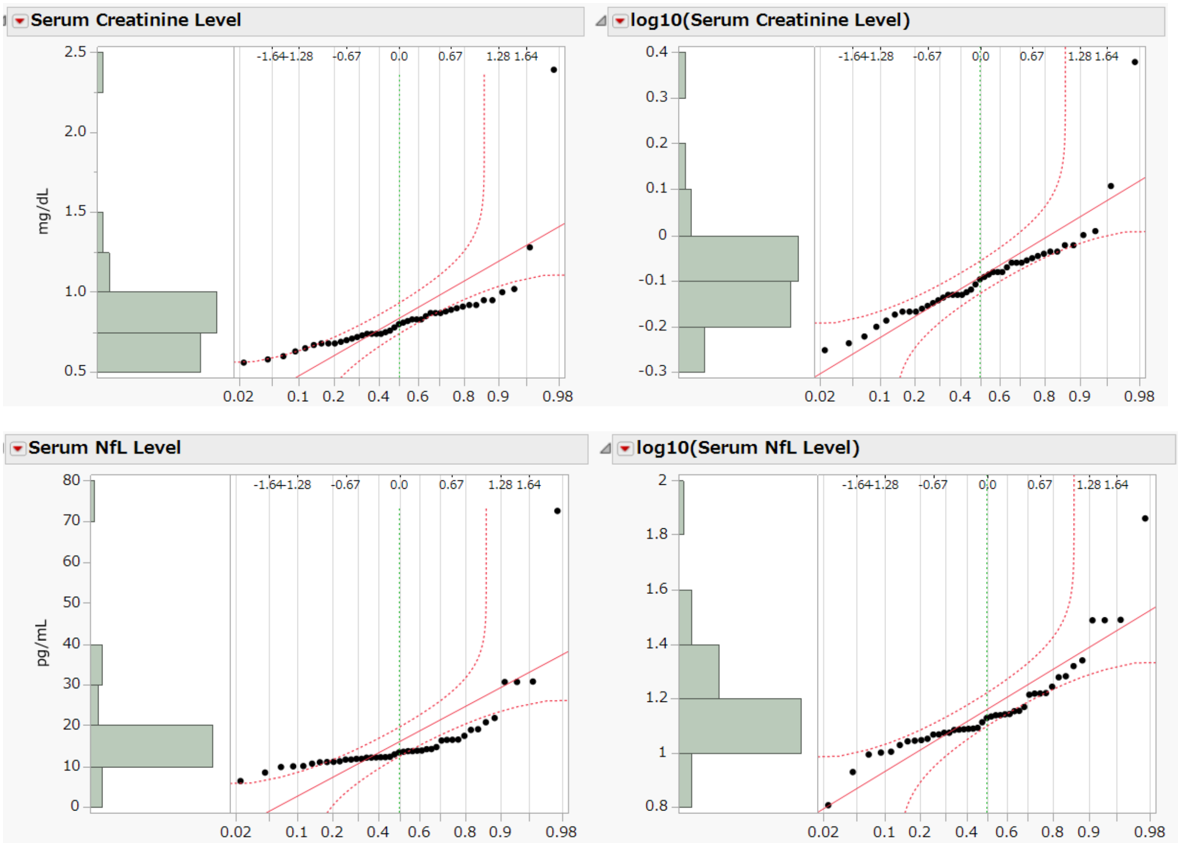
Serum Creatinine Level
mg/dL
log10(Serum Creatinine Level)
Serum NfL Level
pg/mL
log10(Serum NfL Level)
-1.64 -1.28 -0.67 0.0 0.67 1.28 1.64
0.02 0.1 0.2 0.4 0.6 0.8 0.9 0.98
